# Machine learning-based online prediction of nocturnal hypoglycemia in elderly patients with type 2 diabetes

**DOI:** 10.3389/fendo.2025.1685969

**Published:** 2026-01-07

**Authors:** Yuntong Liu, Chenhua Guo, Xinyu Li, Shen Li, Jiajun Huang, Liang Zhao, Yan Zhu, Xuhan Liu, Bing Wang, Rui Lin, Jingshi Wang, Zhengnan Gao, Jing Gao, Yingshu Liu

**Affiliations:** 1 Department of Endocrinology, Central Hospital of Dalian University of Technology, Dalian, China; 2 China Medical University, Shenyang, China; 3 School of Software Technology, Dalian University of Technology, Dalian, China; 4 Department of Central Laboratory, Central Hospital of Dalian University of Technology, Dalian, China; 5 Department of Radiology, Dalian Women and Children’s Medical Group, Dalian, China

**Keywords:** nocturnal hypoglycemia, type 2 diabetes, ensemble learning, elderly people, clinical prediction model

## Abstract

**Context:**

Nocturnal hypoglycemia (NH) is a common adverse event in elderly patients with type 2 diabetes (T2D). This study aims to develop a clinically applicable model for predicting the risk of NH in elderly patients with T2D.

**Methods:**

This retrospective cohort study, conducted from May 2018 to June 2024, analyzed 1,128 elderly T2D patients undergoing continuous glucose monitoring, with an independent validation involving 100 outpatients. Clinical characteristics were collected, and feature engineering was performed to select a manageable set of clinically accessible features. An ensemble model was developed using multiple base models and a stacking approach. The best-performing model was deployed as an online risk calculator.

**Results:**

Of the development set, 288 (25.5%) experienced NH, while 40 (40%) of the independent validation cohort experienced NH. The final ensemble model, “RF-ET-KNN”, combined random forest, Extra Trees, and K-nearest neighbor as base learners, with Extra Trees serving as the meta-learner. It incorporated eleven clinical features and achieved an AUROC of 0.926 and sensitivity of 0.853 on the test set, and an AUROC of 0.947 and sensitivity of 0.929 on the internal validation set. SHAP analysis identified that daytime lowest blood glucose (BG), fasting blood glucose (FBG), and daytime hypoglycemia events were closely related to NH. A user-friendly calculator is available at http://122.51.219.102:8000/.

**Conclusion:**

The “RF-ET-KNN” model, integrating eleven clinically accessible features, effectively predicts NH in elderly T2D patients. Daytime lowest BG, FBG, and daytime hypoglycemia events were significant risk factors.

## 1 Background

With an aging population, the prevalence of diabetes among the elderly is rising. In Western countries, more than 20% of individuals over the age of 65 have diabetes (1, 2). In China, the average prevalence among those aged 60 and older is also above 20% (3). Nocturnal hypoglycemia (NH) is a severe complication in elderly patients with type 2 diabetes (T2D), with its prevalence ranging from 39% to 45% (4, 5). In elderly patients with T2D, the threshold for experiencing hypoglycemic symptoms is lower compared to normal blood glucose levels, which may more easily lead to cognitive dysfunction (6). Consequently, symptoms of NH are frequently obscured and challenging for clinicians to detect. NH can impact multiple organs including the heart, nervous system, and kidneys (7–10); and in severe cases, may lead to bradyarrhythmias, cardiac arrest, and even death (7, 11, 12). Therefore, accurate prediction and mitigation of NH episodes are essential. Continuous glucose monitoring (CGM) offers precise and all-day glucose tracking, facilitating more accurate assessment of nocturnal asymptomatic hypoglycemia.

Recent advancements in machine learning technology have facilitated the application of these techniques in hypoglycemia prediction research, where it has shown promising predictive capabilities. Despite the scarcity of studies focusing on NH in elderly patients with T2D, two notable studies employing multivariate logistic regression (LR) models have emerged (13, 14). In one such study, data derived from CGM showed that daytime mean absolute glucose and pre-midnight mean glucose were the most reliable predictors of NH, achieving a prediction accuracy of 0.756, with a sensitivity of 84% and a specificity of 62.1% (4). These studies, however, did not utilize machine learning approaches and were not translated into clinically applicable models.

Although LR models have played an important role in hypoglycemia prediction, recent advances in deep learning methods have shown substantial improvements in predicting hypoglycemic events. Compared to traditional logistic regression, deep learning methods are capable of handling more complex data features, such as time-series data and higher-dimensional inputs. They offer stronger nonlinear modeling capabilities, higher accuracy, greater stability, and better generalization, while exhibiting a lower risk of overfitting. Consequently, machine learning models often demonstrate superior predictive performance in practical applications. A previous study developed a LSTM model for mild and severe hypoglycemia prediction using a large data set of 619 patients with diabetes from China and the United States (15). The selected features in this study included sex, age, type of diabetes, hemoglobin A1c (HbA1c) levels, and CGM data. Although the LSTM model achieved an AUROC ranging from 0.93 to 0.97 in the validation set, it lacked a clinically applicable implementation. Currently, there are no studies based on machine learning methods to predict NH in elderly patients with T2D. The novelty of this study lies in the combination of machine learning methods and the use of clinically interpretable features, addressing the limitations of traditional approaches in handling complex data and translating these models into clinical applications.

We aimed to develop a novel ensemble learning prediction model that integrates multiple base learners and a meta-learner to accurately assess the risk of NH in elderly patients with T2D. The model utilizes extensive clinical data and CGM indices, including daytime blood glucose levels, to predict nighttime glucose risk. Additionally, we created an online calculator for clinicians to support accurate clinical assessments and treatment decisions, mitigating associated complications of NH.

## 2 Materials and methods

### 2.1 Study subjects

This study analyzed data from 2,455 patients with T2D treated at the Department of Endocrinology and underwent continuous glucose monitoring (CGM), Central Hospital of Dalian University of Technology, between May 2018 and June 2024. Eligible participants were aged ≥ 65 years, diagnosed with T2D based on the 1999 WHO criteria. To address discrepancies between dynamic and actual glucose levels, CGM data from 5 AM to 7 AM was compared with fasting plasma glucose (FPG) concentrations: inclusion criteria required that patients with dynamic glucose ≥ 3.9 mmol/L had to have a fluctuation range of ≤ 20%, while those with dynamic glucose < 3.9 mmol/L required a range of ± 0.83 mmol/L. Exclusion criteria were severe liver or kidney dysfunction and a psychiatric history. Ultimately, 1,128 patients were included in the analysis. For independent validation, 112 elderly outpatient participants with T2D were recruited to wear CGM devices from November 2024 to January 2025, of whom 100 were ultimately included in the validation analysis. Among these 100 participants, 40 (40%) experienced nocturnal hypoglycemia (NH). The study was conducted in accordance with the Declaration of Helsinki and received approval from the Ethics Committee of the Central Hospital of Dalian University of Technology. Informed consent was obtained from outpatient participants, while it was waived for the retrospective inpatient data due to the study’s retrospective nature.

### 2.2 Data collection

Data were collected via the hospital’s electronic medical record system, encompassing antidiabetic medication categories and personal information (Age, height, weight, body mass index [BMI], blood pressure, waist circumference [WC], etc.), biochemical tests (liver function, renal function, lipid profile, FPG, HbA1c, glycated serum albumin, thyroid function, etc.), and assessments of pancreatic function (venous plasma glucose [PG], insulin, and C-peptide measured during a standardized meal test at 0, 0.5, and 2 hours), along with medication usage records. Additionally, dynamic glucose data were recorded every 15 minutes using Abbott FreeStyle Libre CGM system.

### 2.3 Variable definition

The primary outcome variable, NH, was defined as a continuous dynamic glucose level below 3.9 mmol/L for more than 15 minutes during the night (21:00 to 06:00 the following day) (16). In addition to the original variables, five new variables were designed to facilitate a more detailed analysis of hypoglycemia in type 2 diabetes patients: (1) Daytime hypoglycemia: This was defined as at least one instance when dynamic glucose data fell below 3.9 mmol/L during daytime hours (06:00 to 21:00); (2) Daytime highest blood glucose (BG): This recorded the highest glucose level of the day if nocturnal hypoglycemia occurred. If nocturnal hypoglycemia did not occur, it calculated the average of the highest glucose levels each day throughout the study period; (3) Daytime lowest BG: This captured the lowest glucose level of the day when NH was present. If NH was absent, it computed the average of the daily lowest glucose levels during the study period; (4) Glucose fluctuation range: This represented the difference between the highest and lowest daytime glucose levels; (5) Fasting blood glucose (FBG): This was measured as the glucose level recorded from 06:00:00 to 06:14:59 using dynamic glucose data.

The Machine learning models are trained to predict a binary label which is equal to 1 if the patient experienced NH and to 0 otherwise.

### 2.4 Model construction

#### 2.4.1 Data preprocessing

Given the initially low glucose levels at the onset of monitoring and the frequent adjustments in treatment plans, the dynamic glucose records from the first three days of each patient’s data were excluded to enhance the quality of the research analysis. For clinical data with missing entries, missing patterns were investigated, and missing values were imputed using joint modeling inference and multiple imputation techniques. Additionally, collinearity analysis was conducted to identify and exclude features with high correlations to ensure the integrity of the statistical findings.

#### 2.4.2 Dataset partitioning

The dataset was randomly divided into training, testing, and validation sets in a 7:2:1 ratio. The training and testing sets were split 1,000 times for model training and performance evaluation, while the validation set was used to verify model performance. An independent validation set comprising 100 participants was also employed to further evaluate the generalizability of the model.

#### 2.4.3 Feature engineering

Multi-stage feature selection, which comprises mixed feature selection (MFS) and ensemble feature selection (EFS), was designed to select a subset of meaningful, relevant and clinically accessible features for improving the performance of the machine-learning model. The model of MFS is shown in **Supplementary Figure 1**. MFS employed a variety of techniques including forward sequential selection, backward sequential search, Backout, and random sequential search. We evaluated the performance using the extreme gradient boosting (XGBoost) model through cross-validation (CV). The EFS method incorporated eight algorithms—based on median values, Pearson’s product moment correlation test (P_cor), Spearman’s rank correlation test (S_cor), beta-values of LR, error-rate-based variable importance measure (ER_RF), Gini-index-based variable importance measure (Gini_RF), area under the curve-based variable importance measure (AUC_CF), and error-rate-based variable importance measure for cross-fold validation (ER_CF).

#### 2.4.4 Construction of ensemble learning prediction model

**Figure 1:**
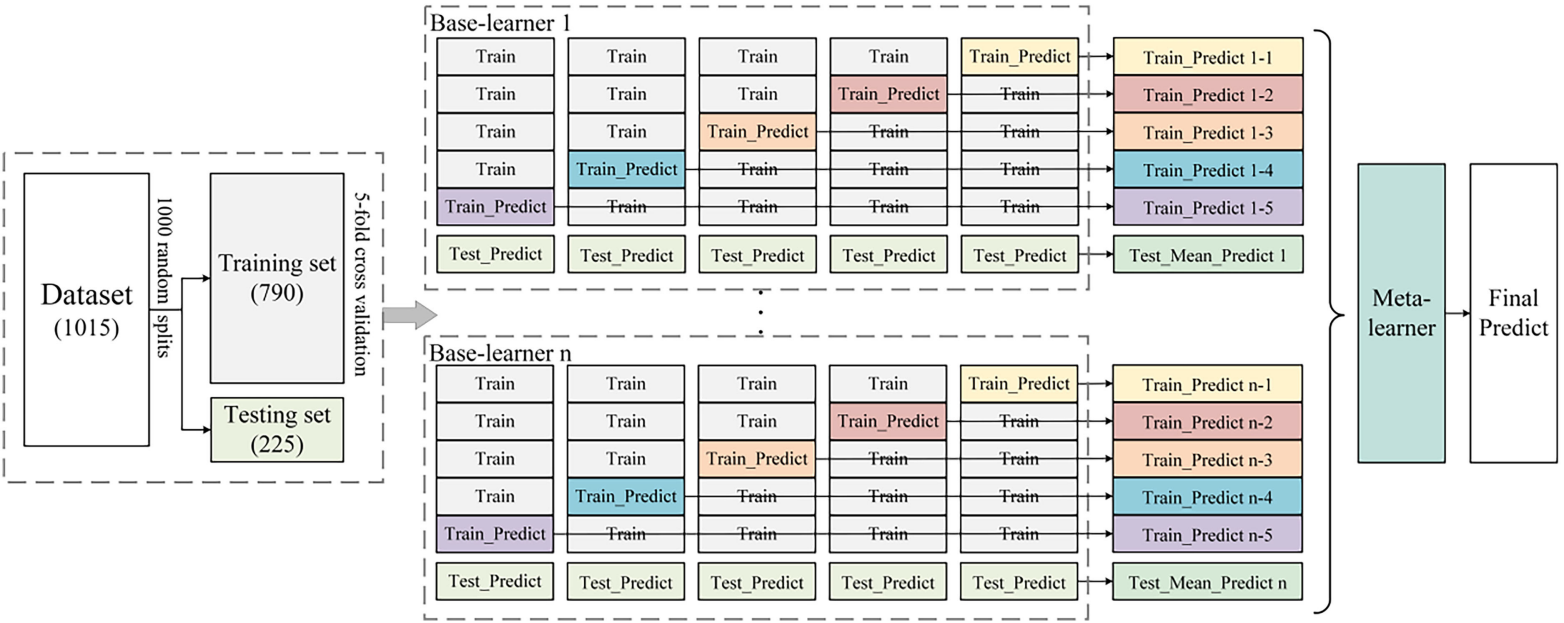
Ensemble learning prediction model. The dataset (N = 1015) is randomly split into a training set (N = 790) and a testing set (N = 225) through 1000 iterations. Within the training set, 5-fold cross-validation is performed to train multiple base-learners (1 to n). Each base-learner is fitted on the 4 parts of the training set and predictions are made for the remaining part (represented as “Train_Predict”). The base-learner also generates predictions on testing set (represented as “Test_Predict”) and the mean predictions from the cross-validation folds for each base-learner are aggregated (represented as “Test_Mean Predict”). The meta-learner uses intermediate predictions (composed of “Train_Predict” and “Test_Mean Predict”) built by multiple base-learners as input to make a final prediction.

The ensemble learning prediction model was constructed using a stacking approach, which combines the outputs of multiple base learners to improve prediction accuracy and robustness. Base learners, selected from models including random forest (RF), gradient boosting decision tree (GBDT), extremely randomized trees (ET), K nearest neighbor (KNN), XGBoost, and SVM were trained on the dataset. The three highest-performing models were selected as base learners. A meta-learner, chosen from the best-performing models, integrated their predictions to generate the final output. The optimal combination of base learners and meta-learner constituted the final ensemble model, as shown in **Figure 1**.

Model parameters were adjusted in the training set, and performance evaluation metrics such as area under the receiver operating characteristic curve (AUROC), accuracy, sensitivity, specificity, F1 Score (F1), area under the precision recall curve (AUPRC), negative predictive value (NPV), and positive predictive value (PPV) were used for evaluation on the testing set. The hyperparameters were tuned through the grid search strategy to obtain the best classification results. This ensemble learning model was ultimately presented in the form of an online calculator.

#### 2.4.5 Model evaluation

The performance of the ensemble learning prediction model was evaluated on both the testing and validation sets by plotting the receiver operating characteristic (ROC) curve. Clinical decision analysis (DCA) was utilized to assess the clinical benefits, measuring the actual efficacy of different models based on decision costs and benefits. Calibration curves were employed to compare the predicted probabilities of the models with the actual observed outcomes, evaluating prediction accuracy. Additionally, Shapley Additive Explanations (SHAP) dependence values were used to illustrate the relationship between each feature in the prediction model and the outcome.

### 2.5 Statistical analyses and model configuration

For continuous variables, normality was assessed using the Kolmogorov-Smirnov test. Variables following a normal distribution were expressed as mean ± standard deviation, and group comparisons were performed using the t-test. For variables that deviated from a normal distribution, the median and interquartile range were used for description, with the Mann-Whitney U test applied for group comparisons. Categorical variables were described using frequencies and percentages, with intergroup comparisons performed via the chi-square test. All statistical tests were carried out using R version 4.0.0 and Python 3.6.13. The machine learning algorithms were implemented using Python 3.8 along with libraries such as “sklearn.ensemble”, “sklearn.model_selection”, and “sklearn.metrics” for model development and evaluation.

## 3 Results

### 3.1 Data preprocessing results

The pattern of missing data was illustrated in **Supplementary Figure 2**, where each column represented a specific missing pattern. The number at the top of each column indicated the number of patients with that specific pattern, while the number at the bottom indicated the number of missing features. The right side of the row represented the name of the feature, and the left side showed the number of patients with missing data for those features. The feature collinearity correlation matrix heatmap was depicted in **Supplementary Figure 3**, where color variations in each cell represented the magnitude of the Pearson correlation coefficients between variables. Following collinearity analysis, and considering the rates of missing data and the ease of clinical application, eight features (weight, height, glucose, insulin, and C-peptide measurements at 0.5 and 2 hours) were excluded. Concurrently, five new features were introduced, culminating in a final selection of 43 features for the model.

### 3.2 Baseline characteristics

The baseline characteristics of the study population are shown in **Supplementary Table 1**, including a total of 1,128 elderly patients with type 2 diabetes, of which 288 (25.5%) were in the NH group. The NH group had a higher proportion of Man, Daytime_Hypoglycemia, Daytime_BG_Fluctuations, while exhibiting lower levels of AGI_Use, Triglycerides, Uric Acid, FBG, Total_Protein, free triiodothyronine (FT3), 0h_PG, 0h_C-peptide, 0h_Insulin, Daytime_Highest_BG, Daytime_Lowest_BG compared to the non-NH group (*P* < 0.05).

### 3.3 Feature engineering experiments and analysis

#### 3.3.1 MFS method

Following mixed feature selection, the optimal combination of features was identified, comprising: Daytime_Lowest_BG, FBG, Daytime_Hypoglycemia, Uric_Acid, 0h_PG, Daytime_BG_Fluctuations, Daytime_Highest_BG, Total Protein, 0h_C-peptide, WC, GA, Triglycerides, FT3, 0h_Insulin, Gender, Drinking, AGI_Use, BMI, Insulin_Use, Age, Creatinine, HbA1c, and SU_or_Glinides_Use.

#### 3.3.2 EFS method

**Figure 2:**
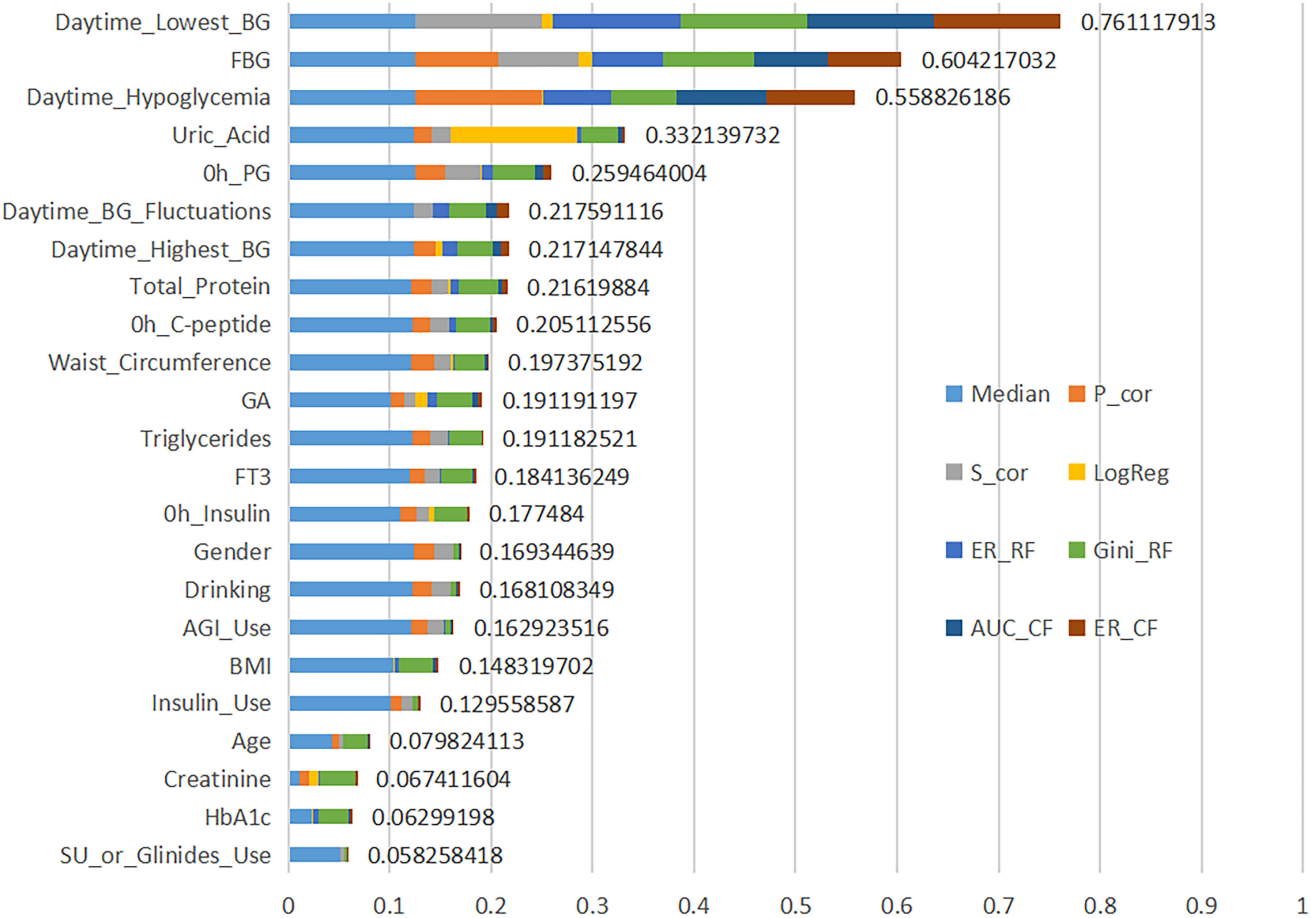
Variables ranking based on EFS score. The y-axis shows the 23 variables ordered by importance value. The x-axis shows the cumulative importance values, calculated via an ensemble of feature selection methods including Median, Pearson’s product moment correlation test (P_cor), Spearman’s rank correlation test (S_cor), beta-values of Logistic regression (LogReg), error-rate-based variable importance measure (ER_RF), Gini-index-based variable importance measure (Gini_RF), area under the curve-based variable importance measure (AUC_CF), and error-rate-based variable importance measure for cross-fold validation (ER_CF). BG, blood glucose; FBG, fasting blood glucose; PG, plasma glucose; GA, glycated albumin; FT3, free triiodothyronine; AGI, alpha-glucosidase inhibitor; BMI, body mass index; HbA1c, hemoglobin A1c; SU, sulfonylurea.

The EFS scores for the features, identified through MFS and the analysis of previously reported risk factors as well as baseline data, were calculated, as shown in **Figure 2**. Based on the EFS results and clinical accessibility, eleven features were ultimately selected for inclusion in the model study: Daytime_Lowest_BG, FBG, Daytime_Hypoglycemia, Daytime_Highest_BG, WC, Gender, Drinking, AGI_Use, BMI, Insulin_Use and Age.

### 3.4 Selection of base learners and meta-learner

The selected features were entered into six foundational models, and the maximum and average values of various model evaluation metrics were calculated from 1,000 random splits of the dataset, as shown in **Supplementary Table 2**. The final candidate base learners included RF (average AUROC: 0.886), ET (average AUROC: 0.905), and KNN (average AUROC: 0.877). Among these, ET, which achieved the highest AUROC, was selected as the meta-learner.

### 3.5 Selection of ensemble learning prediction models

We developed four distinct ensemble learning prediction models: “RF-ET” which combines RF and Extra Trees; “RF-KNN” which pairs RF with KNN; “ET-KNN” which integrates Extra Trees and KNN; and “RF-ET-KNN” which utilizes RF, Extra Trees, and KNN as base learners. In all four models, Extra Trees functioned as the meta-learner. As shown in **Table 1**, the four proposed ensemble learning models outperformed traditional regression analysis methods (LR and Lasso) across all performance metrics. The “RF-ET-KNN” model achieved an average AUROC of 0.926. Predictions with probabilities below 0.5 were classified as non-occurrence of NH, and those equal to or above 0.5 as occurrence of NH.

**Table 1:** Performance comparison of four ensemble learning models on the test set.

Performance Metric	RF-ET	RF-KNN	ET-KNN	RF-ET-KNN	LR	Lasso
Maximum AUROC	0.957	0.960	0.955	0.961	0.915	0.913
Average AUROC	0.918	0.921	0.913	0.926	0.855	0.855
Maximum Accuracy	0.894	0.908	0.884	0.901	0.848	0.842
Average Accuracy	0.832	0.836	0.822	0.840	0.770	0.765
Maximum Sensitivity	0.934	0.941	0.934	0.940	0.895	0.888
Average Sensitivity	0.842	0.849	0.847	0.853	0.778	0.768
Maximum Specificity	0.921	0.921	0.901	0.914	0.861	0.861
Average Specificity	0.821	0.822	0.798	0.826	0.763	0.761
Maximum F1 Score	0.897	0.907	0.889	0.904	0.855	0.849
Average F1 Score	0.833	0.838	0.826	0.842	0.772	0.765
Maximum AUPRC	0.956	0.962	0.952	0.962	0.916	0.915
Average AUPRC	0.921	0.926	0.915	0.929	0.844	0.844
Maximum PPV	0.910	0.912	0.891	0.905	0.847	0.846
Average PPV	0.826	0.828	0.808	0.832	0.767	0.763
Maximum NPV	0.921	0.927	0.925	0.936	0.883	0.876
Average NPV	0.839	0.845	0.840	0.850	0.775	0.767

RF, random forest; ET, extremely randomized trees; KNN, K nearest neighbor; LR, Logistic regression; AUROC, area under the receiver operating characteristic curve; AUPRC, area under the precision recall curve; NPV, negative predictive value; PPV, positive predictive value.

The evaluation results of the four ensemble models on the internal and independent validation set were presented in **Supplementary Table 3** and **Supplementary Table 4**. The final selected model, “RF-ET-KNN” outperformed the other models across most metrics, demonstrating superior predictive performance.

As shown in **Supplementary Table 4**, on the independent validation set, the model achieved an AUROC of 0.874, sensitivity of 0.800, and specificity of 0.782, confirming its robustness and generalizability.

### 3.6 Online calculator

In this study, an online calculator was developed to serve as an interface for clinicians utilizing the model. The “RF-ET-KNN” model was selected for integration into the calculator. During use, clinicians are required to input the patient’s Daytime_lowest_BG, FBG, Daytime_Hypoglycemia, Daytime_highest_BG, BMI, WC, drinking, AGI_use, Insulin_Use, Age, and Gender. The model processed these inputs in the background and displayed the results on the webpage, as illustrated at http://122.51.219.102:8000/.

### 3.7 Evaluation of the prediction model

#### 3.7.1 ROC curve

**Figure 3:**
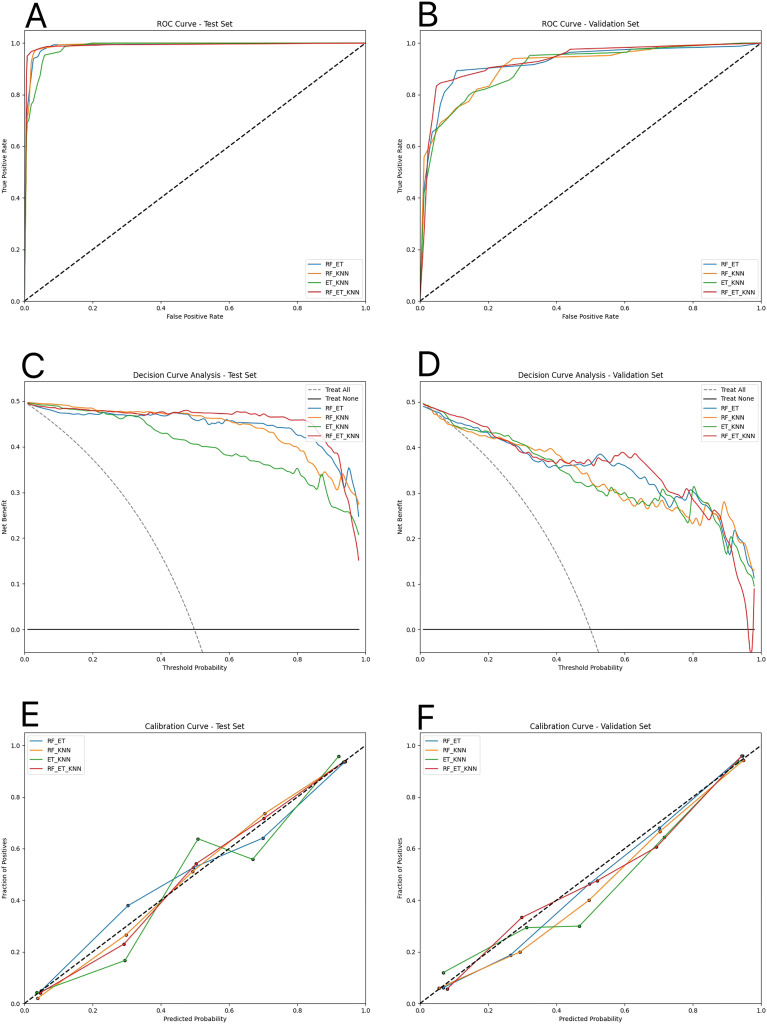
Performance evaluation of four ensemble learning models. **(A, B)** ROC curves of the four models on the test and validation sets, respectively, showing their discrimination performance. **(C, D)** Decision curve analysis (DCA) curves on the test and validation sets, respectively, illustrating the clinical net benefit across various threshold probabilities. **(E, F)** Calibration curves on the test and validation sets, respectively, demonstrating the agreement between predicted and observed outcomes. RF, random forest; ET, extremely randomized trees; KNN, K nearest neighbor.

(**Figures 3A, B** showed that the “RF-ET-KNN” ensemble learning prediction model achieved a high true positive rate and a low false positive rate, demonstrating its robust classification performance.

#### 3.7.2 DCA curve

The DCA illustrated the clinical benefit of each model at various thresholds; a higher curve indicated greater clinical utility. (**Figures 3C, D**) showed that the “RF-ET-KNN” ensemble learning prediction model consistently outperformed other models across the 0.5-0.8 threshold range.

#### 3.7.3 Calibration curve

The calibration curve compared the model’s predicted probabilities (x-axis) with the actual proportion of positive cases (y-axis), with the diagonal line representing ideal calibration. (**Figures 3E, F**) showed that the “RF-ET-KNN” ensemble learning prediction model exhibited better calibration, aligning closely with the ideal diagonal line.

#### 3.7.4 SHAP dependence

**Figure 4:**
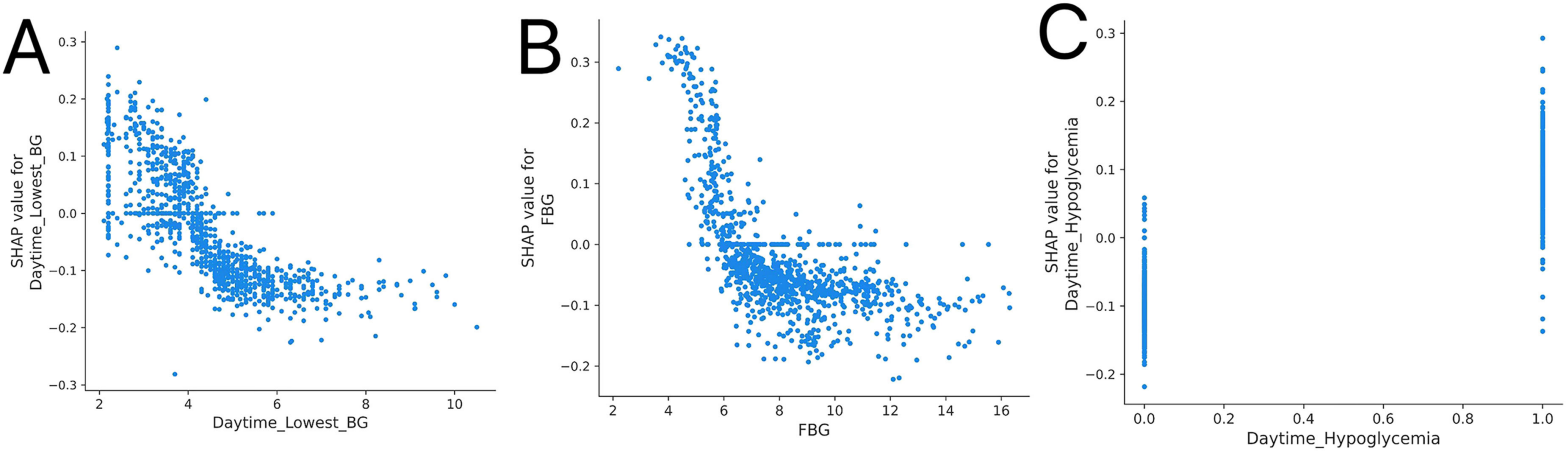
SHAP dependence plots for key predictors associated with NH. **(A)** SHAP dependence plot for daytime lowest BG, **(B)** SHAP dependence plot for FBG, and **(C)** SHAP dependence plot for daytime hypoglycemia, highlighting their relationships with NH. SHAP, Shapley additive explanations; Daytime lowest BG, daytime lowest blood glucose; FBG, fasting blood glucose; NH, nocturnal hypoglycemia.

(**Figures 4A, B**) illustrated that Daytime_lowest_BG and FBG were negatively correlated with the occurrence of NH, whereas Daytime_Hypoglycemia events in (**Figure 4C**) were positively correlated. NH occurrence decreased when Daytime_lowest_BG exceeded 4.5 mmol/L or FBG exceeded 6.5 mmol/L. The absence of Daytime_Hypoglycemia served as a protective factor against the occurrence of NH.

## 4 Discussion

Since NH in elderly patients with T2D is often asymptomatic and easily overlooked, early identification and intervention are of critical importance. Li et al. applied LR, SVM, RF, and LSTM networks to predict future NH events from CGM data in a large cohort of 1,921 patients with diabetes (17). Their study further revealed that combining mean sensor glucose with glucose gradient improved the prediction of NH events, achieving good predictive performance. However, the prediction of NH in their study was based on static analysis and lacked a clinically applicable implementation. In contrast, our study constructed an ensemble model combining RF, ET, and KNN algorithms, integrating CGM data with easily accessible clinical features. Furthermore, we developed a web-based calculator to facilitate clinical application and assist clinicians in decision-making (18). Our work aimed to establish a more accurate and practical model for predicting NH in elderly patients with T2D. The model achieved an average AUROC of 0.947 and sensitivity of 0.929 on the internal validation set, outperforming traditional LR models without CGM (AUROC 0.7–0.75) in predicting NH among elderly T2D patients (13, 14). This finding suggests that the selected data features and the constructed model exhibits superior predictive performance and generalization capability.

The final model is presented as an online calculator for clinical use, providing a foundation for clinical decision-making. In clinical practice, the online calculator can be easily applied during routine visits. Clinicians can simply enter several common clinical variables and daytime glucose measurements to obtain an estimated risk of NH. This provides an early warning that helps to identify high-risk elderly patients with T2D and supports timely adjustment of treatment strategies. Compared with existing tools, few models specifically predict NH in elderly T2D patients. These models are all based on LR and generally show only moderate predictive performance (AUROC 0.7–0.75) (14). Moreover, none have been translated into clinically applicable tools. Our calculator addresses this critical gap by providing a practical and user-friendly solution for clinical implementation. However, certain challenges remain, including the occurrence of false positives and false negatives. The model demonstrated good sensitivity (0.929), which minimizes the likelihood of missing patients at elevated risk for NH. This enables clinicians to initiate timely interventions, thereby reducing adverse outcomes and improving patient safety.

According to the EFS analysis, the three most important features were Daytime_lowest_BG, FBG, and Daytime_Hypoglycemia. The results from the SHAP model indicated that the occurrence of NH decreased when Daytime_lowest_BG exceeded 4.5 mmol/L or when FBG exceeded 6.5 mmol/L. Furthermore, the absence of Daytime_Hypoglycemia was identified as a protective factor against the occurrence of NH.

Over 90% of diabetes cases in the elderly are classified as T2D. The incidence of hypoglycemia in older adults is higher than in younger individuals due to decreased autonomic reflex regulation, slower drug metabolism, reduced treatment adherence, and unstable dietary intake (6, 19). These mechanisms clarify how decreased FBG contributes to lower daytime glucose levels and why Daytime_Hypoglycemia events represent significant risk factors for NH in elderly T2D patients. Firstly, excessive use of insulin or insulin secretagogues can result in absolute insulin excess, while increased nighttime insulin sensitivity may lead to relative insulin excess, both contributing to lowered blood glucose levels. Secondly, excess insulin inhibits alpha cells, reducing glucagon’s response to hypoglycemia, which in turn decreases glucagon secretion and exacerbates blood glucose decline (14, 20, 21). Additionally, recurrent hypoglycemia can impair or eliminate the glucose counter-regulatory mechanism, resulting in decreased secretion of glucose-elevating hormones (such as glucagon and epinephrine) and reduced hepatic glucose production (22–24). This creates a vicious cycle of recurrent hypoglycemia (25, 26). Therefore, addressing these factors is crucial for the prevention and management of hypoglycemic events in older diabetes patients.

The findings of this study align with existing research on risk factors for hypoglycemia. Kanazawa et al. identified FBG, Age, and Insulin_Use as independent risk factors for clinically significant nighttime hypoglycemia in elderly patients with T2D through LR analysis, with FBG being the most significant predictor (13). Similarly, Fang et al. established FBG as a valid predictive indicator for NH in elderly male T2D patients using LR (14). Another study employing LR to predict NH in T2D patients found that Daytime_Hypoglycemia events were also significant predictive factors (4). Notably, we are the first to identify Daytime_lowest_BG levels as an important risk factor for NH in elderly T2D patients. Furthermore, Zale et al.’s research, which utilized random forest methods to predict hypoglycemia in hospitalized patients, corroborates our findings by indicating that the lowest blood glucose level within 24 hours was a significant predictor of hypoglycemia (27).

Several additional features were identified for analysis, including anthropometric indicators (BMI and WC), lifestyle factors (Drinking), demographic characteristics (Age and Gender), and medical interventions (Insulin_Use and AGI_Use). Lower BMI and WC have been associated with an increased risk of hypoglycemia (28–30), likely reflecting poor nutritional status and reduced glycogen stores (31). Underweight patients may also exhibit decreased insulin secretion, requiring higher doses of hypoglycemic medications and increasing NH events (32). Drinking has been linked to hypoglycemic episodes in T2D patients (28, 29), as alcohol inhibits hepatic glycogenolysis and gluconeogenesis, reducing blood glucose production. Furthermore, drinking often correlates with unhealthy lifestyle behaviors, such as irregular eating and lack of exercise, which further contribute to hypoglycemia risk (33). Our findings also align with research demonstrating that hypoglycemia risk increases with age in elderly T2D patients, potentially due to age-related declines in counter-regulatory responses and hepatic glucose production (13, 34). Additionally, similar to this study found that male gender may increase the risk of NH in elderly patients with type T2D (35). Insulin, with its potent glucose-lowering effects, has been consistently associated with hypoglycemic episodes in T2D patients, further supporting our results (13, 36–38). AGI, a glucose-lowering drug, delays carbohydrate absorption by inhibiting α-glucosidase, which helps control blood glucose and, when used alone, does not increase the risk of hypoglycemia (39, 40). The previous study reported no cases of hypoglycemia in 93 elderly patients with T2D treated with AGI (41).

### 4.1 Limitations of the study

Firstly, our study was a single-center investigation and has not yet been externally validated in other populations. Future research should validate the model in diverse populations across various countries and regions to better evaluate its generalization. Secondly, this study did not account for multiple factors such as diet, physical activity, and medication adherence, which could potentially influence NH. However, considering that nighttime food intake and physical activity is typically minimal, the direct impact of dietary carbohydrate intake on nocturnal blood glucose fluctuations is likely limited, although not entirely negligible. Future studies should consider incorporating these variables into the model to improve prediction accuracy and to better understand their contribution to the risk of NH.

## 5 Conclusion

This study developed a well-performing and generalizable ensemble learning model based on “RF-ET-KNN” utilizing CGM data and clinical indicators to identify 11 clinically accessible risk factors. The model was further implemented as an online calculator for clinical use.

## Data Availability

The raw data supporting the conclusions of this article will be made available by the authors, without undue reservation.

## References

[1] Saelee R Hora IA Pavkov ME Imperatore G Chen Y Benoit SR . Diabetes prevalence and incidence inequality trends among U.S. Adults, 2008-2021. Am J Prev Med. (2023) 65:973–82. doi: 10.1016/j.amepre.2023.07.009, PMID: 37467866 PMC10792096

[2] Magliano DJ Boyko EJ . IDF diabetes atlas. Jama. (2022) 326:2498–2506.

[3] Wang L Peng W Zhao Z Zhang M Shi Z Song Z . Prevalence and treatment of diabetes in China, 2013-2018. Jama. (2021) 326:2498–506. doi: 10.1001/jama.2021.22208, PMID: 34962526 PMC8715349

[4] Klimontov VV Myakina NE . Glucose variability indices predict the episodes of nocturnal hypoglycemia in elderly type 2 diabetic patients treated with insulin. Diabetes Metab syndrome. (2017) 11:119–24. doi: 10.1016/j.dsx.2016.08.023, PMID: 27569727

[5] Ishikawa T Koshizaka M Maezawa Y Takemoto M Tokuyama Y Saito T . Continuous glucose monitoring reveals hypoglycemia risk in elderly patients with type 2 diabetes mellitus. J Diabetes Invest. (2018) 9:69–74. doi: 10.1111/jdi.12676, PMID: 28397367 PMC5754529

[6] Bellary S Kyrou I Brown JE Bailey CJ . Type 2 diabetes mellitus in older adults: clinical considerations and management. Nat Rev Endocrinology. (2021) 17:534–48. doi: 10.1038/s41574-021-00512-2, PMID: 34172940

[7] Andersen A Jørgensen PG Knop FK Vilsbøll T . Hypoglycaemia and cardiac arrhythmias in diabetes. Ther Adv Endocrinol Metab. (2020) 11:2042018820911803. doi: 10.1177/2042018820911803, PMID: 32489579 PMC7238305

[8] Yun JS Park YM Han K Kim HW Cha SA Ahn YB . Severe hypoglycemia and the risk of end stage renal disease in type 2 diabetes. Sci Rep. (2021) 11:4305. doi: 10.1038/s41598-021-82838-5, PMID: 33619302 PMC7900096

[9] Yaffe K Falvey CM Hamilton N Harris TB Simonsick EM Strotmeyer ES . Association between hypoglycemia and dementia in a biracial cohort of older adults with diabetes mellitus. JAMA Internal Med. (2013) 173:1300–6. doi: 10.1001/jamainternmed.2013.6176, PMID: 23753199 PMC4041621

[10] Punthakee Z Miller ME Launer LJ Williamson JD Lazar RM Cukierman-Yaffee T . Poor cognitive function and risk of severe hypoglycemia in type 2 diabetes: *post hoc* epidemiologic analysis of the ACCORD trial. Diabetes Care. (2012) 35:787–93. doi: 10.2337/dc11-1855, PMID: 22374637 PMC3308284

[11] Novodvorsky P Bernjak A Chow E Iqbal A Sellors L Williams S . Diurnal differences in risk of cardiac arrhythmias during spontaneous hypoglycemia in young people with type 1 diabetes. Diabetes Care. (2017) 40:655–62. doi: 10.2337/dc16-2177, PMID: 28213374

[12] Chow E Bernjak A Williams S Fawdry RA Hibbert S Freeman J . Risk of cardiac arrhythmias during hypoglycemia in patients with type 2 diabetes and cardiovascular risk. Diabetes. (2014) 63:1738–47. doi: 10.2337/db13-0468, PMID: 24757202

[13] Kanazawa K Suzuki S Koga S Kuwabara K . A comprehensive risk assessment for nocturnal hypoglycemia in geriatric patients with type 2 diabetes: A single-center case-control study. J Diabetes its complications. (2022) 36:108239. doi: 10.1016/j.jdiacomp.2022.108239, PMID: 35810146

[14] Fang F Xiao H Li C Tian H Li J Li Z . Fasting glucose level is associated with nocturnal hypoglycemia in elderly male patients with type 2 diabetes. Aging male. (2013) 16:132–6. doi: 10.3109/13685538.2013.818111, PMID: 23876123

[15] Shao J Pan Y Kou WB Feng H Zhao Y Zhou K . Generalization of a deep learning model for continuous glucose monitoring-based hypoglycemia prediction: algorithm development and validation study. JMIR Med informatics. (2024) 12:e56909. doi: 10.2196/56909, PMID: 38801705 PMC11148841

[16] Danne T Nimri R Battelino T Bergenstal RM Close KL DeVries JH . International consensus on use of continuous glucose monitoring. Diabetes Care. (2017) 40:1631–40. doi: 10.2337/dc17-1600, PMID: 29162583 PMC6467165

[17] Li J Ma X Tobore I Liu Y Kandwal A Wang L . A novel CGM metric-gradient and combining mean sensor glucose enable to improve the prediction of nocturnal hypoglycemic events in patients with diabetes. J Diabetes Res. (2020) 2020:8830774. doi: 10.1155/2020/8830774, PMID: 33204733 PMC7655247

[18] Mahajan P Uddin S Hajati F Moni MA . Ensemble learning for disease prediction: A review. Healthcare (Basel Switzerland). (2023) 11. doi: 10.3390/healthcare11121808, PMID: 37372925 PMC10298658

[19] Silbert R Salcido-Montenegro A Rodriguez-Gutierrez R Katabi A McCoy RG . Hypoglycemia among patients with type 2 diabetes: epidemiology, risk factors, and prevention strategies. Curr Diabetes Rep. (2018) 18:53. doi: 10.1007/s11892-018-1018-0, PMID: 29931579 PMC6117835

[20] Cryer PE . Severe iatrogenic hypoglycemia in type 2 diabetes mellitus. Nat Clin Pract Endocrinol Metab. (2007) 3:4–5. doi: 10.1038/ncpendmet0355, PMID: 17179922

[21] Paranjape SA Chan O Zhu W Horblitt AM McNay EC Cresswell JA . Influence of insulin in the ventromedial hypothalamus on pancreatic glucagon secretion *in vivo*. Diabetes. (2010) 59:1521–7. doi: 10.2337/db10-0014, PMID: 20299468 PMC2874714

[22] Taborsky GJ Jr. Mundinger TO . Minireview: The role of the autonomic nervous system in mediating the glucagon response to hypoglycemia. Endocrinology. (2012) 153:1055–62. doi: 10.1210/en.2011-2040, PMID: 22315452 PMC3384078

[23] Cryer PE . Minireview: Glucagon in the pathogenesis of hypoglycemia and hyperglycemia in diabetes. Endocrinology. (2012) 153:1039–48. doi: 10.1210/en.2011-1499, PMID: 22166985 PMC3281526

[24] Ly TT Hewitt J Davey RJ Lim EM Davis EA Jones TW . Improving epinephrine responses in hypoglycemia unawareness with real-time continuous glucose monitoring in adolescents with type 1 diabetes. Diabetes Care. (2011) 34:50–2. doi: 10.2337/dc10-1042, PMID: 20929999 PMC3005473

[25] Orban BO Routh VH Levin BE Berlin JR . Direct effects of recurrent hypoglycaemia on adrenal catecholamine release. Diabetes Vasc Dis Res. (2015) 12:2–12. doi: 10.1177/1479164114549755, PMID: 25268022 PMC8771481

[26] Dagogo-Jack SE Craft S Cryer PE . Hypoglycemia-associated autonomic failure in insulin-dependent diabetes mellitus. Recent antecedent hypoglycemia reduces autonomic responses to, symptoms of, and defense against subsequent hypoglycemia. J Clin Invest. (1993) 91:819–28. doi: 10.1172/JCI116302, PMID: 8450063 PMC288033

[27] Zale AD Abusamaan MS McGready J Mathioudakis N . Development and validation of a machine learning model for classification of next glucose measurement in hospitalized patients. EClinicalMedicine. (2022) 44:101290. doi: 10.1016/j.eclinm.2022.101290, PMID: 35169690 PMC8829081

[28] Yun JS Han K Choi SY Cha SA Ahn YB Ko SH . External validation and clinical application of the predictive model for severe hypoglycemia. Front endocrinology. (2022) 13:1006470. doi: 10.3389/fendo.2022.1006470, PMID: 36246915 PMC9556834

[29] Han K Yun JS Park YM Ahn YB Cho JH Cha SA . Development and validation of a risk prediction model for severe hypoglycemia in adult patients with type 2 diabetes: a nationwide population-based cohort study. Clin Epidemiol. (2018) 10:1545–59. doi: 10.2147/CLEP.S169835, PMID: 30425585 PMC6203120

[30] Chow LS Zmora R Ma S Seaquist ER Schreiner PJ . Development of a model to predict 5-year risk of severe hypoglycemia in patients with type 2 diabetes. BMJ Open Diabetes Res Care. (2018) 6:e000527. doi: 10.1136/bmjdrc-2018-000527, PMID: 30116541 PMC6091902

[31] Arvidsson Kvissberg ME Hu G Chi L Bourdon C Ling C ChenMi Y . Inhibition of mTOR improves malnutrition induced hepatic metabolic dysfunction. Sci Rep. (2022) 12:19948. doi: 10.1038/s41598-022-24428-7, PMID: 36402829 PMC9675758

[32] Funakoshi S Fujimoto S Hamasaki A Fujiwara H Fujita Y Ikeda K . Analysis of factors influencing pancreatic beta-cell function in Japanese patients with type 2 diabetes: association with body mass index and duration of diabetic exposure. Diabetes Res Clin practice. (2008) 82:353–8. doi: 10.1016/j.diabres.2008.09.010, PMID: 18950889

[33] Yun JS Han K Park YM Han E Lee YH Ko SH . Adherence to healthy lifestyle behaviors as a preventable risk factor for severe hypoglycemia in people with type 2 diabetes: A longitudinal nationwide cohort study. J Diabetes Invest. (2022) 13:1533–42. doi: 10.1111/jdi.13818, PMID: 35474300 PMC9943249

[34] Shi M Yang A Lau ESH Luk AOY Ma RCW Kong APS . A novel electronic health record-based, machine-learning model to predict severe hypoglycemia leading to hospitalizations in older adults with diabetes: A territory-wide cohort and modeling study. PLoS Med. (2024) 21:e1004369. doi: 10.1371/journal.pmed.1004369, PMID: 38607977 PMC11014435

[35] Mathioudakis NN Everett E Routh S Pronovost PJ Yeh HC Golden SH . Development and validation of a prediction model for insulin-associated hypoglycemia in non-critically ill hospitalized adults. BMJ Open Diabetes Res Care. (2018) 6:e000499. doi: 10.1136/bmjdrc-2017-000499, PMID: 29527311 PMC5841507

[36] Kronborg T Hangaard S Hejlesen O Vestergaard P Jensen MH . Bedtime prediction of nocturnal hypoglycemia in insulin-treated type 2 diabetes patients. J Diabetes Sci technology. (2024) 18:592–7. doi: 10.1177/19322968221141736, PMID: 36514195 PMC11089861

[37] Karter AJ Warton EM Lipska KJ Ralston JD Moffet HH Jackson GG . Development and validation of a tool to identify patients with type 2 diabetes at high risk of hypoglycemia-related emergency department or hospital use. JAMA Internal Med. (2017) 177:1461–70. doi: 10.1001/jamainternmed.2017.3844, PMID: 28828479 PMC5624849

[38] Crutzen S Belur Nagaraj S Taxis K Denig P . Identifying patients at increased risk of hypoglycaemia in primary care: Development of a machine learning-based screening tool. Diabetes/metabolism Res Rev. (2021) 37:e3426. doi: 10.1002/dmrr.3426, PMID: 33289318 PMC8518928

[39] Agrawal N Sharma M Singh S Goyal A . Recent advances of α-glucosidase inhibitors: A comprehensive review. Curr topics medicinal Chem. (2022) 22:2069–86. doi: 10.2174/1568026622666220831092855, PMID: 36045528

[40] Cai X Yang W Zhou L Zhang S Han X Ji L . Comparisons of the efficacy of glucose control, lipid profile, and β-cell function between DPP-4 inhibitors and AGI treatment in type 2 diabetes patients: a meta-analysis. Endocrine. (2015) 50:590–7. doi: 10.1007/s12020-015-0653-3, PMID: 26048437

[41] Josse RG Chiasson JL Ryan EA Lau DC Ross SA Yale JF . Acarbose in the treatment of elderly patients with type 2 diabetes. Diabetes Res Clin practice. (2003) 59:37–42. doi: 10.1016/S0168-8227(02)00176-6, PMID: 12482640

