# Supplementary material for: Machine learning-based online prediction of nocturnal hypoglycemia in elderly patients with type 2 diabetes
Source: Front Endocrinol (Lausanne). 2026 Jan 7;16:1685969. doi: 10.3389/fendo.2025.1685969 (PMC12819298; doi:10.3389/fendo.2025.1685969)
Supplement: Supplementary file 1 [file DataSheet1.docx]

**Supplementary file**

Yuntong Liu, Chenhua Guo, Xinyu Li, Shen Li, Jiajun Huang, Liang Zhao, Yan Zhu, Xuhan Liu, Bing Wang, Rui Lin, Jingshi Wang, Zhengnan Gao, Jing Gao, and Yingshu Liu

Machine Learning-Based Online Prediction of Nocturnal Hypoglycemia in Elderly Patients with Type 2 Diabetes

**eTable 1.** Baseline characteristics between the two groups.

**eTable 2.** Performance comparison of six machine learning models.

**eTable 3.** Performance comparison of four ensemble learning prediction models on the internal validation set.

**eTable 4.** Performance comparison of four ensemble learning prediction models on the independent validation set.

**eFigure 1.** Multi-stage feature selection flowchart.

**eFigure 2.** Exploration of patient missing data patterns.

**eFigure 3.** Feature collinearity correlation matrix heatmap.

This supplementary file has been provided by the authors to give readers additional information about their work.

**eTable 1.** Baseline characteristics between the two groups.

| Variables | non-NH  (N=840) | NH  (N=288) | *P* values |
| --- | --- | --- | --- |
| Gender (%) |  |  | 0.0075 |
| Man | 388 (46.2%) | 160 (55.6%) |  |
| Woman | 452 (53.8%) | 128 (44.4%) |  |
| Age (years) | 71.0 (67.0, 76.0) | 70.5 (68.0, 75.0) | 0.8272 |
| Drinking (%) | 192 (22.9%) | 80 (28.0%) | 0.1020 |
| Smoking (%) | 544 (64.9%) | 174 (60.6%) | 0.2172 |
| SBP (mmHg) | 135.0 (120.75, 146.0) | 135.0 (120.0, 148.0) | 0.8794 |
| DBP (mmHg) | 80.0 (74.0, 86.0) | 80.0 (75.0, 88.0) | 0.4346 |
| BMI (Kg/m^2^) | 25.1 (22.8, 27.1) | 25.0 (22.25, 26.45) | 0.1971 |
| Waist_Circumference (cm) | 94.57 ± 9.87 | 93.22 ± 8.90 | 0.0533 |
| Glucose lowering drugs (%) |  |  |  |
| Insulin_Use | 608 (72.7%) | 199 (71.1%) | 0.6464 |
| GLP-1RA_Use | 333 (39.8%) | 106 (37.9%) | 0.6066 |
| Metformin _Use | 491 (58.7%) | 173 (61.8%) | 0.4062 |
| AGI_Use | 494 (59.1%) | 144 (51.4%) | 0.0298 |
| TZD_Use | 7 (0.8%) | 0 (0.0%) | 0.2719 |
| SU_or Glinides_Use | 55 (6.6%) | 17 (6.1%) | 0.8739 |
| SGLT2i_Use | 119 (14.2%) | 37 (13.2%) | 0.7440 |
| DPP-4i_Use | 75 (9.0%) | 28 (10.0%) | 0.6925 |
| TC (mmol/L) | 4.49 ± 1.14 | 4.41 ± 1.13 | 0.3097 |
| LDL (mmol/L) | 2.83 ± 1.05 | 2.78 ± 1.05 | 0.4921 |
| HDL (mmol/L) | 1.0 (0.8525, 1.19) | 1.02 (0.86, 1.25) | 0.1453 |
| TG (mmol/L) | 1.455 (1.06, 1.98) | 1.26 (0.93, 1.89) | 0.0014 |
| ALT (U/L) | 19.0 (13.875, 27.0) | 17.75 (14.0, 28.5) | 0.7205 |
| AST (U/L) | 18.0 (15.0, 23.0) | 18.75 (15.0, 24.0) | 0.3294 |
| UA (umol/L) | 314.0 (260.0, 378.25) | 299.5(248.25, 360.75) | 0.0423 |
| Creatinine (umol/L) | 58.5 (49.0, 72.0) | 58.225 (49.0, 71.75) | 0.7907 |
| Urea (mmol/L) | 6.33 (5.2925, 7.64) | 6.23 (5.16, 7.83) | 0.7501 |
| FBG (mmol/L) | 8.12 (6.82, 9.78) | 6.54 (5.54, 8.07) | <0.001 |
| GA (%) | 23.65 (19.8, 29.2) | 25.0 (19.0, 32.05) | 0.1651 |
| HbA1c (%) | 9.0 (7.5, 10.7) | 9.15 (7.6, 10.9) | 0.4474 |
| Albumin (g/L) | 39.8(37.825, 42) | 39.7 (37.125, 42.2) | 0.7589 |
| TP (g/L) | 66.73 ± 5.48 | 65.86 ± 6.25 | 0.0297 |
| FT3 (mmol/L) | 4.678 (4.31, 5.04) | 4.58 (4.1975, 4.96) | 0.0332 |
| FT4 (mmol/L) | 15.12 (13.75, 16.92) | 15.44 (13.85, 17.33) | 0.0972 |
| TSH (mIU/L) | 1.79(1.165, 2.79) | 1.773(1.12, 2.718) | 0.6747 |
| Potassium_Ion (mmol/L) | 4.04 ± 0.35 | 4.03 ± 0.37 | 0.9570 |
| Sodium_Ion (mmol/L) | 139.6(138.2, 141.0) | 139.7 (138.41, 141.2) | 0.2483 |
| 0h_C-peptide (nmol/L) | 1.25(0.82, 1.745) | 1.08 (0.702, 1.73) | 0.0158 |
| 0h_Insulin (μU/mL) | 11.9 (7.5, 20.5) | 11.0 (6.975, 17.7) | 0.0359 |
| 0h_PG (mmol/L) | 8.19 (6.7, 10.32) | 7.38 (5.86, 9.86) | <0.001 |
| Daytime_Highest_BG (mmol/L) | 15.7 (13.2, 18.83) | 14.9 (12.28, 18.23) | 0.0063 |
| Daytime_Lowest_BG (mmol/L) | 4.7 (3.88, 5.53) | 3.4 (2.8, 3.9) | <0.001 |
| Daytime_BG_Fluctuations (mmol/L) | 11.1 (8.4, 14.3) | 11.55 (8.80, 14.7) | 0.0337 |
| Daytime_Hypoglycemia (%) | 210 (25.0%) | 215 (74.7%) | <0.001 |

Data are expressed as mean (P25, P75) or number (percent). Abbreviation: NH, nocturnal hypoglycemia; SBP, systolic blood pressure; DBP, diastolic blood pressure; BMI, body mass index; GLP-1RA, glucagon-like peptide-1 receptor agonists; AGI, alpha-glucosidase inhibitor; TZD, thiazolidinediones; SU, sulfonylurea; SGLT2i, sodium-glucose cotransporter 2 inhibitors; DPP-4i, dipeptidyl peptidase-4 inhibitors; HDL, high-density lipoprotein; LDL, low-density lipoprotein; TC, cholesterol; TG, triglyceride; ALT, alanine aminotransferase; AST, aspartate aminotransferase; UA, uric acid; FBG, fasting blood glucose; GA, glycated serum albumin; HbA1c, hemoglobin A1c; TP, total protein; FT3, free triiodothyronine; FT4, free thyroxine; TSH, thyroid-stimulating hormone; PG, Plasma Glucose.

**eTable 2.** Performance comparison of six machine learning models.

|  | RF | ET | KNN | GBDT | XGBoost | SVM |
| --- | --- | --- | --- | --- | --- | --- |
| Maximum AUROC | 0.933 | 0.947 | 0.932 | 0.918 | 0.918 | 0.912 |
| Average AUROC | 0.886 | 0.905 | 0.877 | 0.869 | 0.873 | 0.856 |
| Maximum Accuracy | 0.861 | 0.889 | 0.857 | 0.846 | 0.843 | 0.836 |
| Average Accuracy | 0.788 | 0.806 | 0.791 | 0.776 | 0.777 | 0.755 |
| Maximum Sensitivity | 0.915 | 0.915 | 0.977 | 0.884 | 0.891 | 0.853 |
| Average Sensitivity | 0.787 | 0.809 | 0.880 | 0.768 | 0.769 | 0.747 |
| Maximum Specificity | 0.881 | 0.921 | 0.861 | 0.887 | 0.901 | 0.868 |
| Average Specificity | 0.788 | 0.803 | 0.716 | 0.782 | 0.785 | 0.762 |
| Maximum F1 Score | 0.852 | 0.876 | 0.858 | 0.834 | 0.835 | 0.817 |
| Average F1 Score | 0.774 | 0.793 | 0.795 | 0.759 | 0.761 | 0.737 |
| Maximum AUPRC | 0.848 | 0.902 | 0.837 | 0.843 | 0.864 | 0.837 |
| Average AUPRC | 0.761 | 0.779 | 0.726 | 0.751 | 0.754 | 0.729 |
| Maximum PPV | 0.914 | 0.919 | 0.973 | 0.882 | 0.887 | 0.856 |
| Average PPV | 0.813 | 0.832 | 0.875 | 0.799 | 0.800 | 0.779 |
| Maximum NPV | 0.924 | 0.944 | 0.932 | 0.903 | 0.912 | 0.906 |
| Average NPV | 0.869 | 0.893 | 0.844 | 0.847 | 0.853 | 0.831 |

Abbreviation: RF, random forest; ET, [extremely randomized trees](https://www.baidu.com/s?sa=re_dqa_generate&wd=Extremely%20Randomized%20Trees&rsv_pq=d92b63640009e23f&oq=ExtraTrees%E5%85%A8%E7%A7%B0&rsv_t=7845/9uSJEnQ5FoXcqL81dBTnYhpbda4Epi2K9XHk8jJHGJgI7IxMHy6e4poJVV7M+3Mg5s&tn=25017023_17_dg&ie=utf-8); KNN, K nearest neighbor; GBDT, gradient boosting decision tree; XGBoost, [extreme gradient boosting](https://www.baidu.com/s?wd=eXtreme%20Gradient%20Boosting&rsv_idx=2&tn=25017023_17_dg&usm=4&ie=utf-8&rsv_pq=e3d5c0a50015e95f&oq=XGBoost%E5%85%A8%E7%A7%B0&rsv_t=5583856iUypucQj4iLFfSGwTofifSMmaEG4DvxGlJeNyFmRwMvrHWNLHV0n7aMNCoOIo48k&sa=re_dqa_generate); SVM, support vector Machine; AUROC, area under the receiver operating characteristic curve; AUPRC, area under the precision recall curve; NPV, negative predictive value; PPV, positive predictive value.

**eTable 3.** Performance comparison of four ensemble learning prediction models on the internal validation set.

|  | RF-ET | RF-KNN | ET-KNN | RF-ET-KNN |
| --- | --- | --- | --- | --- |
| AUROC | 0.937 | 0.923 | 0.923 | 0.947 |
| Accuracy | 0.839 | 0.839 | 0.804 | 0.851 |
| Sensitivity | 0.917 | 0.905 | 0.869 | 0.929 |
| Specificity | 0.762 | 0.774 | 0.738 | 0.774 |
| F1 Score | 0.851 | 0.849 | 0.816 | 0.862 |
| AUPRC | 0.947 | 0.934 | 0.932 | 0.948 |
| PPV | 0.794 | 0.800 | 0.768 | 0.804 |
| NPV | 0.901 | 0.890 | 0.849 | 0.915 |

Abbreviation: RF, random forest; ET, [extremely randomized trees](https://www.baidu.com/s?sa=re_dqa_generate&wd=Extremely%20Randomized%20Trees&rsv_pq=d92b63640009e23f&oq=ExtraTrees%E5%85%A8%E7%A7%B0&rsv_t=7845/9uSJEnQ5FoXcqL81dBTnYhpbda4Epi2K9XHk8jJHGJgI7IxMHy6e4poJVV7M+3Mg5s&tn=25017023_17_dg&ie=utf-8); KNN, K nearest neighbor; AUROC, area under the receiver operating characteristic curve; AUPRC, area under the precision recall curve; NPV, negative predictive value; PPV, positive predictive value.

**eTable 4.** Performance comparison of four ensemble learning prediction models on the independent validation set.

|  | RF-ET | RF-KNN | ET-KNN | RF-ET-KNN |
| --- | --- | --- | --- | --- |
| AUROC | 0.855 | 0.857 | 0.876 | 0.874 |
| Accuracy | 0.782 | 0.764 | 0.791 | 0.791 |
| Sensitivity | 0.709 | 0.745 | 0.745 | 0.800 |
| Specificity | 0.855 | 0.782 | 0.836 | 0.782 |
| F1 Score | 0.765 | 0.759 | 0.781 | 0.793 |
| AUPRC | 0.830 | 0.852 | 0.868 | 0.859 |
| PPV | 0.830 | 0.774 | 0.820 | 0.786 |
| NPV | 0.746 | 0.754 | 0.767 | 0.796 |

Abbreviation: RF, random forest; ET, [extremely randomized trees](https://www.baidu.com/s?sa=re_dqa_generate&wd=Extremely%20Randomized%20Trees&rsv_pq=d92b63640009e23f&oq=ExtraTrees%E5%85%A8%E7%A7%B0&rsv_t=7845/9uSJEnQ5FoXcqL81dBTnYhpbda4Epi2K9XHk8jJHGJgI7IxMHy6e4poJVV7M+3Mg5s&tn=25017023_17_dg&ie=utf-8); KNN, K nearest neighbor; AUROC, area under the receiver operating characteristic curve; AUPRC, area under the precision recall curve; NPV, negative predictive value; PPV, positive predictive value.

**eFigure 1.** Multi-stage feature selection flowchart.


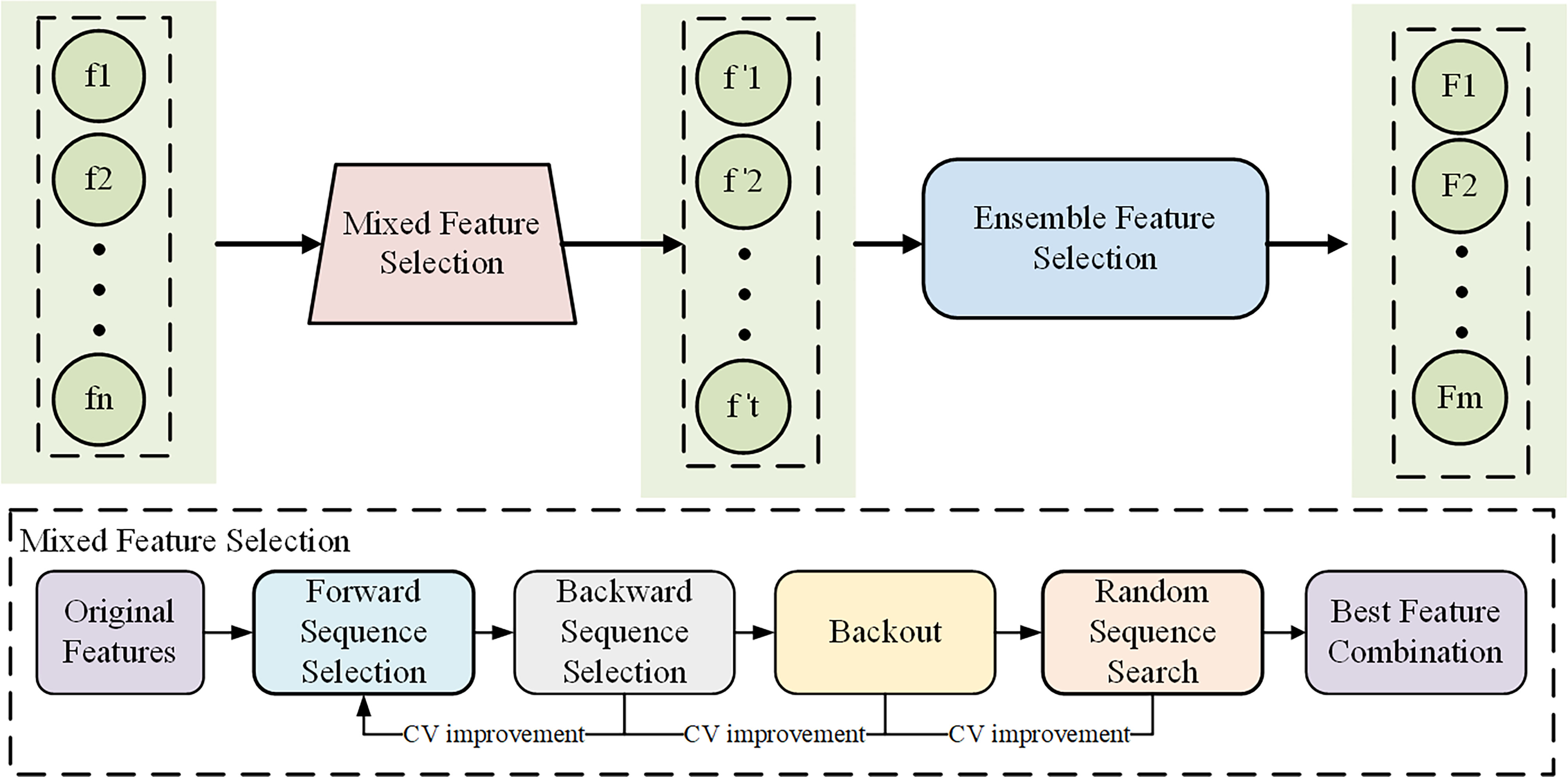


Abbreviation: CV, cross validation.

**eFigure 2.** Exploration of patient missing data patterns.


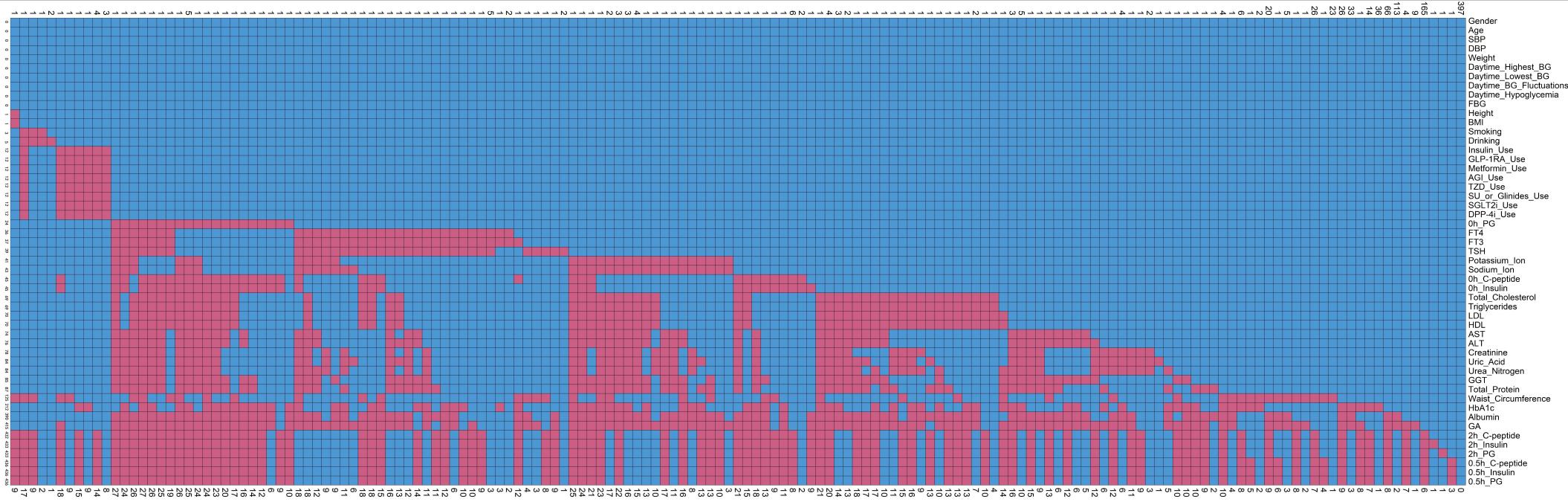


Abbreviation: SBP, systolic blood pressure; DBP, diastolic blood pressure; BMI, body mass index; GLP-1RA, glucagon-like peptide-1 receptor agonists; AGI, alpha-glucosidase inhibitor; TZD, thiazolidinediones; SU, sulfonylurea; SGLT2i, sodium-glucose cotransporter 2 inhibitors; DPP-4i, dipeptidyl peptidase-4 inhibitors; HDL, high-density lipoprotein; LDL, low-density lipoprotein; TC, cholesterol; TG, triglyceride; ALT, alanine aminotransferase; AST, aspartate aminotransferase; UA, uric acid; FBG, fasting blood glucose; GA, glycated serum albumin; HbA1c, hemoglobin A1c; TP, total protein; FT3, free triiodothyronine; FT4, free thyroxine; TSH, thyroid-stimulating hormone; PG, Plasma Glucose.

Each column represents a specific missing pattern. The number at the top of each column indicates the number of patients with that specific pattern, while the number at the bottom indicates the number of missing features. The right side of the row represents the name of the feature, and the left side shows the number of patients with missing data for those features. For example, in the rightmost column, the feature 0.5 h_PG (bottom row) has 436 missing values in total. In the second column from the right, the bottom number 3 indicates that three features, including 0.5h_PG, are missing together. The top number 1 shows that one patient has this specific combination of missing features.

**eFigure 3.** Feature collinearity correlation matrix heatmap.


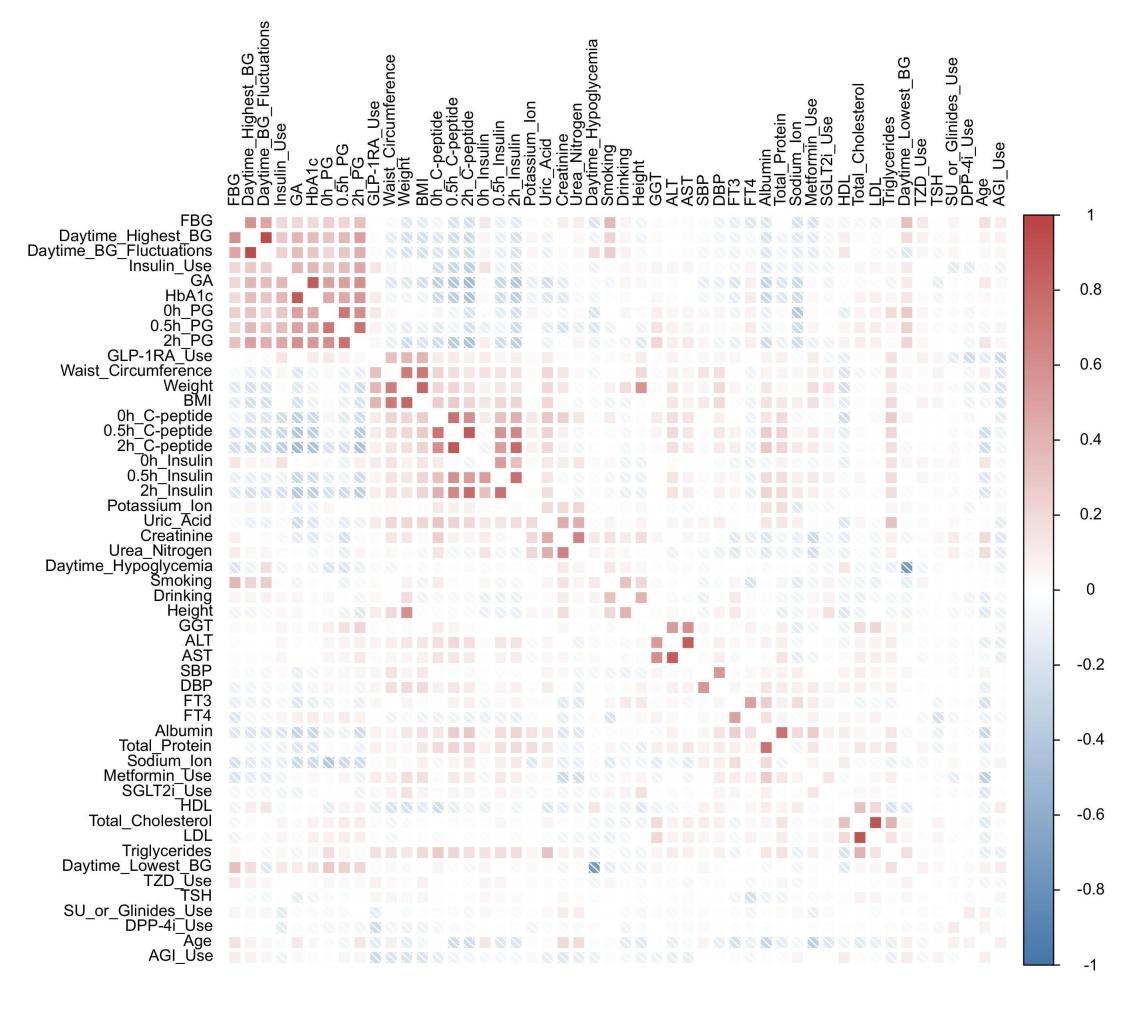


Abbreviation: SBP, systolic blood pressure; DBP, diastolic blood pressure; BMI, body mass index; GLP-1RA, glucagon-like peptide-1 receptor agonists; AGI, alpha-glucosidase inhibitor; TZD, thiazolidinediones; SU, sulfonylurea; SGLT2i, sodium-glucose cotransporter 2 inhibitors; DPP-4i, dipeptidyl peptidase-4 inhibitors; HDL, high-density lipoprotein; LDL, low-density lipoprotein; TC, cholesterol; TG, triglyceride; ALT, alanine aminotransferase; AST, aspartate aminotransferase; UA, uric acid; FBG, fasting blood glucose; GA, glycated serum albumin; HbA1c, hemoglobin A1c; TP, total protein; FT3, free triiodothyronine; FT4, free thyroxine; TSH, thyroid-stimulating hormone; PG, Plasma Glucose.

Color variations in each cell represent the magnitude of the Pearson correlation coefficients between variables.
